# First case of the novel GrOwnValve procedure—a case report

**DOI:** 10.1093/ehjcr/ytag548

**Published:** 2026-07-20

**Authors:** Alexander Breitenstein-Attach, Marvin Steitz, Boris Schmitt

**Affiliations:** Department of Congenital Heart Disease—Pediatric Cardiology, Deutsches Herzzentrum der Charité, Augustenburger Platz 1, Berlin 13353, Germany; Charité—Universitätsmedizin Berlin, Corporate Member of Freie Universität Berlin and Humboldt-Universität zu Berlin, Charitéplatz 1, Berlin 10117, Germany; German Centre for Cardiovascular Research, Potsdamer Str. 58, Berlin 10785, Germany; Department of Congenital Heart Disease—Pediatric Cardiology, Deutsches Herzzentrum der Charité, Augustenburger Platz 1, Berlin 13353, Germany; Charité—Universitätsmedizin Berlin, Corporate Member of Freie Universität Berlin and Humboldt-Universität zu Berlin, Charitéplatz 1, Berlin 10117, Germany; German Centre for Cardiovascular Research, Potsdamer Str. 58, Berlin 10785, Germany; Department of Congenital Heart Disease—Pediatric Cardiology, Deutsches Herzzentrum der Charité, Augustenburger Platz 1, Berlin 13353, Germany; Charité—Universitätsmedizin Berlin, Corporate Member of Freie Universität Berlin and Humboldt-Universität zu Berlin, Charitéplatz 1, Berlin 10117, Germany; German Centre for Cardiovascular Research, Potsdamer Str. 58, Berlin 10785, Germany

Current pulmonary valve replacements rely on foreign xenogenic or synthetic prostheses, which remain prone to structural degeneration and reinterventions^1,2^. Autologous tissue may overcome these limitations, but existing approaches (like the Ross and Ozaki procedures) require complex surgical reconstruction^3,4^. We report the first clinical application of the GrOwnValve procedure, enabling intraoperative manufacturing and transcatheter implantation of a patient-specific pulmonary valve derived from autologous pericardium^4^. The procedure is intended to provide a durable solution and may therefore enable treatment of pediatric patients.

A 35-year-old patient with severe pulmonary homograft stenosis (maximum gradient 115 mmHg) following Ross procedure was enrolled in a prospective first-in-human study. Based on preprocedural computed tomography imaging (*Figure 1A*), patient-specific 3D-printed molds were manufactured for valve fabrication. During the intervention, autologous pericardium was harvested via mini-thoracotomy (*Figure 1B*), prepared, shaped into a patient-specific three-leaflet valve (*Figure 1C, B*), mounted onto a balloon-expandable stent, crimped (*Figure 1E, F*), and implanted via a transcatheter approach into the presented pulmonary homograft (*Figure 1G*). The procedure was completed without complications. At 6-month follow-up, echocardiography demonstrated a reduction in V_max_ from 5.08 to 1.89 m/s, mean/maximum transvalvular gradients from 56/103 to 7/14 mmHg, and an increase in pressure half-time from 158 to 350 ms (*Figure 1H–J*). Cardiac MRI confirmed a reduction in regurgitant fraction from 20% to 10% and right ventricular end-diastolic volume index from 196 to 133 mL/m^2^, indicating reverse ventricular remodeling. This first-in-human application demonstrates the feasibility of intraoperative manufacturing and transcatheter implantation of an autologous pulmonary valve prosthesis. A detailed description is provided in the Supplementary material.

**Figure 1:**
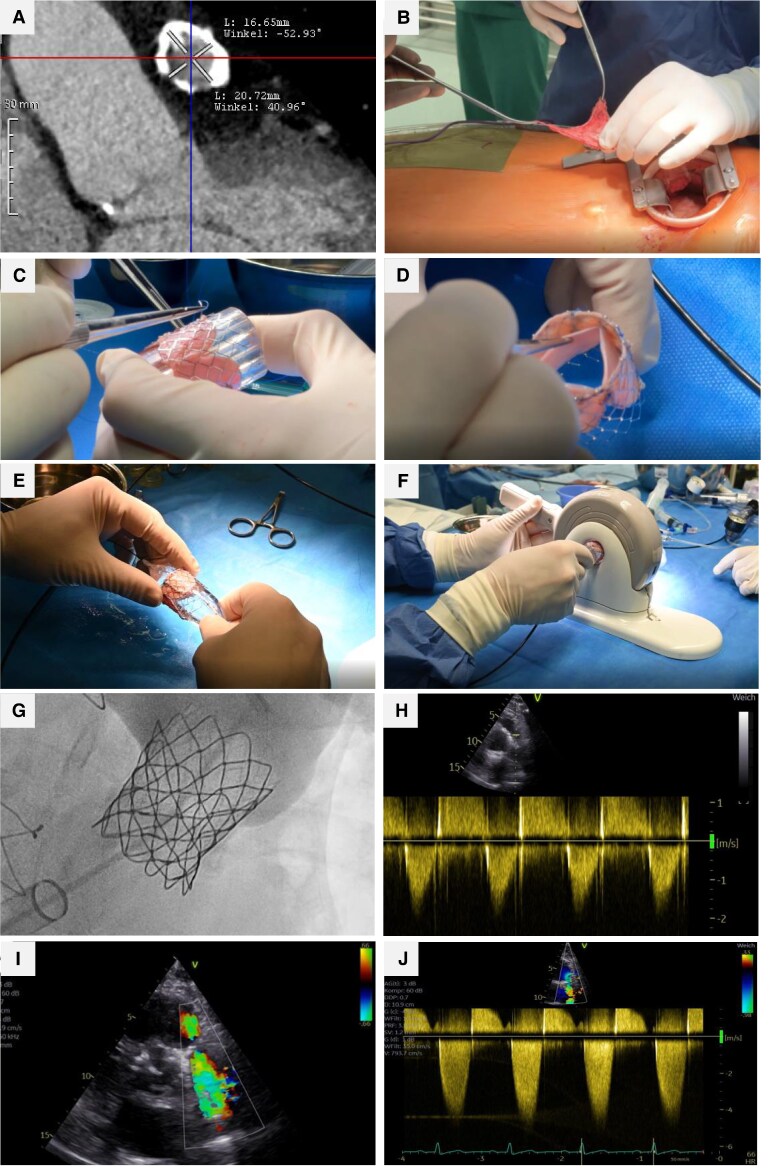
Visualization of GrOwnValve procedure: (*A*) CT-reconstruction and annulus measurement; (*B*) harvesting of pericardium via mini-thoracotomy; (*C*) molding and suturing onto stent frame; (*D*) check for commissure alignment; (*E–F*) crimping and loading onto balloon catheter; (*G*) transcatheter implantation; (*H–J*) echocardiographic assessment after (*H*) and before (*I*, *J*) implantation.

## Supplementary Material

ytag548_Supplementary_Data

## Data Availability

The data that support the findings of this study are available from the corresponding author upon reasonable request. The clinical study ‘GECT’ is conducted in accordance with the principles of the Declaration of Helsinki and in accordance with Section 3, Paragraph 4 MPDG, and Article 82, Paragraph 1 MDR (EU) 2017/74 (ClinicalTrials.gov Identifier: NCT05809856).

## Notes

**Consent:** The authors confirm that written consent for submission and publication of this case report, including image(s) and associated text, has been obtained from the patient in line with COPE guidance.
