# Supplementary material for: First case of the novel GrOwnValve procedure—a case report
Source: Eur Heart J Case Rep. 2026 Jul 20;10(8):ytag548. doi: 10.1093/ehjcr/ytag548 (PMC13426910; doi:10.1093/ehjcr/ytag548)
Supplement: ytag548_Supplementary_Data [file ytag548_supplementary_data.zip › Supplementary_Appendix.docx]

**Appendix**

**Detailed patient description**

A 35-year-old man with a congenital bicuspid aortic valve and valvular aortic stenosis underwent balloon valvuloplasty in 1995. Following the development of progressive aortic regurgitation, a Ross procedure was performed in 2002 with implantation of a pulmonary homograft. During routine follow-up in early 2024, severe degeneration of the pulmonary homograft was detected. Transthoracic echocardiography demonstrated severe pulmonary homograft stenosis with a peak gradient of 115 mmHg and concomitant moderate neo-aortic valve insufficiency. Right ventricular hypertrophy was present, while no signs of overt heart failure were observed (NYHA class I). Cardiac magnetic resonance imaging confirmed severe homograft stenosis (V_max_ 5.08 m/s), mild pulmonary regurgitation (regurgitant fraction 20%), and marked right ventricular dilatation (RVEDVi 196 mL/m²) with preserved systolic function (RV-EF 51%). Severe neo-aortic valve insufficiency (regurgitant fraction 54%) was also detected.

Based on the haemodynamic findings, both pulmonary and aortic valve replacement were indicated. However, the patient declined repeat open-heart surgery and refused implantation of a mechanical prosthesis because of the associated requirement for lifelong anticoagulation. Following informed consent, the patient was enrolled in the prospective first-in-human GrOwnValve feasibility study.

**Detailed procedural description**

Prior to intervention, a dynamic transcatheter aortic valve replacement (TAVR) CT was performed, utilizing a SOMATOM Definition Flash (syngo CT VA48A, Siemens, Munich, Germany). Subsequently, based on the 4D-CT reconstruction process, valve sizing was performed based on transversal, frontal and sagittal plane measurements (*Figure S1*). The dimensions were derived area-based, perimeter-based, as well as under consideration of short and long axis, utilizing the FDA and CE certified reconstruction software OsiriX MD (Pixmeo SARL, Bernex, Switzerland). Due to the planned pre-procedural balloon dilation to reduce stenosis, as well as a required oversizing value for the GrOwnValve implant from 5-20%, a valve size of 26 mm was anticipated for intervention. The respective moulds were manufactured via polyjet 3D-printing utilizing a Stratasys OBJET 30 V5 Prime (Stratasys Ltd, Minneapolis, USA) and biocompatible clear resin MED610 (Stratasys Ltd, Minneapolis, USA).

During the intervention, cardiac catheterization was initially performed, including balloon sizing of the right ventricular outflow tract (RVOT) and pulmonary artery (PA) trunk with a 34 mm Amplatzer sizing balloon (Abbott GmbH & Co. KG, Wiesbaden, Germany). This confirmed a stenotic pulmonary homograft with an annular diameter of approximately 19 mm and an expanded RVOT diameter of approximately 26 mm. Simultaneous coronary angiography excluded coronary artery compression.

Given the presence of a heavily calcified homograft, the PA trunk was first reinforced with a covered CP stent deployed on a 22 mm balloon-in-balloon (BiB) catheter (NuMED Inc., Hopkinton, NY, USA). Due to a remaining notch, sequential post-dilatations were performed with 24 mm and 26 mm Atlas Gold balloons (Bard Inc., New Providence, NJ, USA). A mild residual notch remained at the level of the pulmonary valve annulus, without compromise of the RCA perfusion. Repeated angiographic balloon sizing measured a dilated annulus of 23.8 mm, confirming the anticipated valve size of 26 mm.

Subsequently, a 5x5 cm autologous pericardial patch was harvested through a left-lateral mini-thoracotomy (*Figure S2 A*). Due to extensive adhesions, partial resection was required and facilitated using electrocautery. The harvested tissue was replaced with a GORE-TEX Tissue Patch (W. L. Gore & Associates, Inc., Newark, DE, USA).

Following confirmation of appropriate size (*Figure S2 B*), the patch was cleaned and then shaped into a patient-specific heart valve using the 3D-printed mould (26 mm) and cross-linking solution (*Figure S2 C*). The engineered valve was then sutured onto a 33 mm Optimus XXL stent (Andratec GmbH, Koblenz, Germany) *(Figure S2 D*). Following completion of suturing, the valve leaflets were cut open using the patient-specific cutting tool to ensure a smooth and uniform free edge of the cusps. The mould was then removed (*Figure S2 E*), and the valve, including the suture lines, underwent inspection and was approved for implantation (*Figure S2 F*). The valve prosthesis was crimped onto a 26 mm BiB catheter (Altosa-XL-GEMINI PTA Balloon, Andratec GmbH, Koblenz, Germany) (*Figure S2 G*) using a crimping device (EDWARDS CRIMPER, Edwards Lifesciences, Irvine, CA, USA) (*Figure S2 H*). The crimped valve was advanced through a 26-F Gore DrySeal introducer sheath (W. L. Gore & Associates, Inc., Flagstaff, AZ, USA) and delivered to the predefined landing zone. Deployment of the prosthesis was successfully within the dedicated landing zone given by the pre-stent (*Figure S3*). For further reduction of the pressure gradient, the implanted valve was re-dilated using the previously described 26 mm Atlas Gold balloon. Post-dilatation assessment demonstrated an annulus diameter of 25.5 mm.

**Detailed Follow-Up description**

Intraoperative echocardiography demonstrated a mean transvalvular gradient of 10 mmHg and only mild regurgitation. Following discharge, the patient was followed up after 3 and 6 months, via cardiopulmonary exercise testing, echocardiography, magnetic resonance imaging, and other assessments. Exercise capacity improved from 180 to 164 and 196 W after 3 and 6 months, respectively. Echocardiography showed a sustained reduction in peak transvalvular velocity from 5.08 to 2.00 and 1.89 m/s and an increase in pressure half-time from 158 to 315 and 350 ms after 3 and 6 months, respectively, consistent with preserved valve function throughout follow-up (*Figure S4*). These findings were confirmed by cardiac magnetic resonance imaging (only performed after 6 months), which demonstrated a marked reduction in peak gradient from 115 to 16 mmHg, regurgitant fraction from 20% to 10%, and right ventricular end-diastolic volume index from 196 to 133 mL/m^2^, indicating improved haemodynamics and reverse right ventricular remodelling.

The patient began taking valsartan (160.00 mg daily) one year prior to the procedure, and the dose was halved four months after the procedure. In addition, the patient took 75.00 mg of clopidogrel daily for 10 months post-procedure. Acetylsalicylic acid is taken at a dose of 100.00 mg daily and is to be discontinued approximately 1 year after the procedure.
