# Supplementary material for: First case of the novel GrOwnValve procedure—a case report
Source: Eur Heart J Case Rep. 2026 Jul 20;10(8):ytag548. doi: 10.1093/ehjcr/ytag548 (PMC13426910; doi:10.1093/ehjcr/ytag548)
Supplement: ytag548_Supplementary_Data [file ytag548_supplementary_data.zip › Supplementary_Figures.docx]

Figure S1: CT-Reconstruction and annulus measurement in transversal (A,B), sagittal (C,D) and frontal (E,F) plane during diastolic and systolic phase, respectively (area-based and circumferencial measurement in transversal plane not shown).

Figure S2: GrOwnValve Procedure: A) Harvesting of pericardium via mini-thoracotomy; B) Tissue preparation; C) Moulding into the desired shape; D) Suturing onto the stent frame; E-F) Check for commissure alignment; G)-H) Crimping and loading onto balloon catheter.

Figure S3: GrOwnValve Implantation: A) Pre-Stent implantation; B) Pre-Stent dilation with 26 mm high pressure Atlas Gold balloon, C-D) Angiographic assessment of implanted GrOwnValve systolic and diastolic phase, respectively.

Figure S4: Echocardiographic images at discharge (A,B), 3-month (B,C) and 6-month follow-up (E,F) as well as MRI flow visualization at 6-month follow-up (G,I) during systole and diastole, respectively.
