# Supplementary material for: First case of the novel GrOwnValve procedure—a case report
Source: Eur Heart J Case Rep. 2026 Jul 20;10(8):ytag548. doi: 10.1093/ehjcr/ytag548 (PMC13426910; doi:10.1093/ehjcr/ytag548)
Supplement: ytag548_Supplementary_Data [file ytag548_supplementary_data.zip › Supplementary_Follow-Up_Table.docx]

Table 1: Cardiopulmonary*, echocardiographic** and MRI*** parameters from baseline, discharge up to 6-month Follow-Up

|  | **Baseline** Prior to procedure | **Discharge** | **FU-1** 3 months | **FU-2** 6 months |
| --- | --- | --- | --- | --- |
| **Power [W]*** | 180 | / | 164 | 196 |
| **PV V_max_ [m/s]**** | 5.08 | 2.09 | 2.00 | 1.89 |
| **PV PG_mean/max_ [mmHg]**** | 56/103 | 9/18 | 9/16 | 7/14 |
| **PV PHT [ms]**** | 158 | 285 | 315 | 350 |
| **PV RF [%]***** | 20 | / | / | 10 |
| **RV-EDVi [ml/m^2^]***** | 196 | / | / | 133 |
